# Cognitive determinants of self-care behaviors among patients with heart failure: A path analysis

**DOI:** 10.15171/hpp.2018.39

**Published:** 2018-10-27

**Authors:** Haidar Nadrian, Javad Shojafard, Hassan Mahmoodi, Zeinab Rouhi, Hassan Rezaeipandari

**Affiliations:** ^1^Social Determinants of Health Research Center, Tabriz University of Medical Sciences, Tabriz, Iran; ^2^Food and Drug Association Research Center, Ministry of Health and Medical Education, Tehran, Iran; ^3^Department of Health Education and Promotion, Faculty of Health, Tabriz University of Medical Sciences, Tabriz, Iran

**Keywords:** Heart failure, Self-care behavior, Health Belief Model, Path analysis

## Abstract

**Background:** Heart failure (HF) is a common clinical syndrome resulting from any structural or functional cardiac disorder that impairs the ability of ventricles to fill with or eject blood.Our aim in this study was to examine the possible direct/indirect effects of health belief model(HBM) constructs on self-care behaviors among HF patients.

**Methods:** A secondary analysis was conducted on an HBM-based data set collected from 180 patients with HF who were recruited from a heart hospital in Tehran, Iran, during a prospective experimental study in 2008. A regression-based path analysis was conducted to examine the relationships between HBM constructs (as independent variables) and self-care behaviors (as dependent variable).

**Results:** A conceptual path model was identified for the cognitive determinants of self-care behaviors among HF patients. Knowledge (β = 0.399), perceived barriers (β = 0.315) and susceptibility (β = 0.165) had direct effects on self-care (R2 = 0.512, P < 0.001). Perceived benefits, self-efficacy, severity and threat, locus of control and cues to action had indirect effects on self-care through the first three variables.

**Conclusion:** HBM was found to be helpful in understanding direct and indirect associations between the cognitive determinants and self-care behaviors among HF patients. Based on this challenging path analysis, HF patients’ knowledge and perceived barriers and susceptibility are suggested as the most core categories while designing HF educational programs. Better understanding on such associations may lead nurses and health practitioners in designing properly informed stage-specific educational interventions aiming to foster self-care behaviors among HF patients.

## Introduction

Heart failure (HF) is a common clinical syndrome resulting from any structural or functional cardiac disorder that impairs the ability of ventricles to fill with or eject blood.^1^ It is a major challenge for healthcare providers due to the high rates of mortality and morbidity associated with the disease.^2^ Worldwide, about 1%-2% of adults are suffered from HF, and this rate increases to 6%-10% among adults older than 65.^3^ It has also affected approximately 5.7 million of population in the United States.^4^ In Iran, the number of patients with congestive HF is reported to be 3,337 per 100 000.^5^ The median age of death and the percent of years of life lost (YLL) were 65.7 and 1.7%, respectively.^3^ Compared to individuals without HF, the patients with HF have a 4-fold higher mean of healthcare expenditures.^6^

Nearly half of readmissions among HF patients are considered to be preventable, and poor adherence with the recommended self-care among the patients is identified as a contributing factor.^7^ Self-care education is an important intervention for the management of HF; however, patient education in practice varies considerably.^8^ Having a good understanding on the factors associated with the behavior seems to be necessary while designing appropriate self-care education programs and health promoting behaviors.^9^ For researchers in the field of HF patients’ education, theoretical frameworks may be helpful in investigating the determinants of self-care behaviors in a logical manner. Behavioral models and health theories^10^ provide the investigators with such frameworks. One of the behavioral models that has been widely used to assess the cognitive determinants of health behaviors is the health belief model (HBM).^11^ This model was originally developed in the 1950s, extended in the 1980s^10^ and since then, has been widely used in health education and behavior change studies.^12-15^ Extended HBM is based on the principle that individuals perform a healthy behavior if they feel that they are at risk (perceived susceptibility), the risks of unsafe behavior are serious (perceived severity), the healthy behavior is beneficial for them (perceived benefits), the barriers to healthy behavior can be removed (perceived barriers), and they are able to have healthy behavior (self-efficacy).^10^

Boyde et al in a systematic review on educational interventions for patients with HF concluded that a patient-centered approach to education based on educational theories and with appropriate evaluation may be helpful in developing evidence-based HF patients’ education programs.^16^ Another review of literature in 2010 showed that both short- and long-term interventions can improve self-efficacy among HF patients demonstrating that the duration of an intervention may vary and still be successful.^17^

## Aim

In the present study, we examined the possible relationships between the HBM constructs and self-care behaviors among HF patients. We attempted to identify the pattern of cognitive factors associated to self-care behaviors among the patients. The following questions guided the study:

1. How is the pattern of self-care behaviors among patients with HF in Tehran, Iran?
2. What are the most significant cognitive predictors of self-care behaviors among these patients?
3. How is the pattern of HBM-based cognitive factors associated to self-care behaviors among patients with HF?

## Materials and Methods

### Design

A secondary analysis was conducted on the pre-test data collected from 180 patients with HF who were recruited from a heart hospital in Tehran, Iran, during a prospective experimental study in 2008.^18^ Face-to-face private interviews were conducted in a private room at the hospital for the purpose of data collection. The characteristics of the study and the sample size justification are published elsewhere.^18,19^

### Instrumentation 

The process of instrumentation was explained in the previously published papers.^18,19^ However, in order to better clarify the process, we chose to have a brief explanation. The following procedure was carried out to translate the instruments into Persian. A back-translation technique^20^ was used to achieve a Persian translation, which preserved the denotation and connotation of each of the instruments items. A native English speaker with mastery of the Persian language who had not seen the original English versions of the scales conducted back-translation. The back-translated copies were compared to the original English scales by the investigators to recognize incongruities. The Persian translations were adjusted with corrective re-translation as necessary, prior to use. While developing some scales and translating some other to Persian, various ways of wording questions were considered to avoid the possibility that certain responses may be consistently chosen in error.

An expert panel, consisting 6 scholars in the areas of health behavior and education, a cardiologist and a nurse with field experience in HF, reviewed and assessed the questions, orally, by evaluating the appropriateness and relevance of the items to HF patients, response format and confirm them to be representative of the constructs in order to confirm content validity of the instruments. The feedback from the panel of experts, which mostly was regarding the wording and phrasing of questions, was used to revise and modify the instruments, which were then pilot tested by a sample of 20 HF outpatients to examine their utility. The pilot study was conducted to identify the problems/benefits associated with the design. The first draft was prepared following consultation with the multidisciplinary team. The data were used to estimate the internal consistency of the scales, using Cronbach α. The content validity of the scales was also established. This pilot sample was not included in the final sample. The scales, number of items, reliability coefficients in pilot and final sample, and possible ranges of the constructs are listed in Table 1.

### Statistical analyses

The IBM SPSS Statistics software (version 20; IBM Corp*.,* Chicago*,* IL*,* USA) was used for the purpose of data entry, manipulation, and analysis. In the present path analysis study, measures of central tendency and variability were used to summarize and organize the data. Inferential statistics were used to answer the research questions. Specifically, Spearman rho correlation coefficient, were used. Regression-based path analysis was also performed to investigate the pattern of HBM-based cognitive factors associated to self-care behavior among patients with HF.

As noted by Munro, “path analysis is an analysis of the paths or lines in a model which represents the influence of on variable on another”.^21^ In order to answer the questions on the relationships between the HBM constructs (as independent variables) and self-care behaviors (as dependent variable) a regression-based path analysis was conducted. Such path analysis is based on simple regression techniques and moves the researcher beyond testing the prediction of a phenomenon by a set of dependent variables to investigate the associations among those variables.^21^ The stages of regression-based path analysis in our study were as follow:

At the first step, the variables that had statistically significant correlations with self-care behaviors in the Spearman correlation coefficient test (knowledge, perceived susceptibility, perceived barriers, perceived benefit, severity, perceived threat, locus of control and self-efficacy) were assessed to predict self-care behaviors applying linear regression analysis. At the step 2, the variables that significantly predicted self-care behaviors (knowledge; perceived susceptibility; and perceived barriers) were again regressed on self-care behaviors to determine the strongest predictor for continuing the path. At the step 3, the strongest predictor identified in the previous step (knowledge) was considered as the dependent variable. The other remaining variables of the model, as independent variables, were regressed on knowledge. At the step 4, perceived barrier, as the significant predictor in the previous step, was considered as the dependent variable, and all the other remaining variables of the model, as independent variables, were regressed on perceived barrier. In this step, 2 variables (perceived susceptibility and self-efficacy) were significant predictors of the dependent variable. At the step 5, the stronger predictor in the previous step, self-efficacy, was considered as the dependent variable, and all the other remaining variables of the model, as independent variables, were regressed on self-efficacy. In this step, the only significant predictor was perceived susceptibility.

At the step 6, perceived susceptibility, as the only predictor in the previous step, was considered as the dependent variable, and all the other remaining variables of the model, as independent variables, were regressed on perceived susceptibility. In this step, 2 variables (perceived severity and locus of control) were significant predictors of the dependent variable. At the step 7, locus of control, as the strongest predictor in previous step, was considered as the dependent variable, and all the 4 remaining variables of the model, as independent variables, were regressed on locus of control. In this step, the only significant predictor was perceived severity. At the step 8, perceived severity, as the only predictor in the previous step, was considered as the dependent variable, and all the 3 remaining variables of the model, as independent variables, were regressed on perceived severity. In this step, 2 variables (perceived threat and perceived benefits) were significant predictors of the dependent variable. At the step 9, perceived benefits, as the strongest predictor in previous step, was considered as the dependent variable, and the 2 remaining variables of the model, as independent variables, were regressed on perceived benefits. In this step, the significant predictor was perceived threat. At the step 10, perceived threat, as the only predictor in the previous step, was considered as the dependent variable, and cues to action as the only remaining variable of the model, was regressed on perceived threat. Therefore, the regression-based path analysis terminated in this step.

The standardized Beta values found in the stages number 2 to 10 of regression analysis were considered as path coefficient, which is an estimation of the direct effect of the independent variables on the dependent variable. To determine the indirect effects of independent variables on the dependent variable, the beta values of the indirect paths were multiplied by each other. The total effect of the independent variables on the dependent variable was calculated by summing the total multiplies of the direct and indirect pathways.

## Results

In the present study the data on 180 patients with HF were included in a secondary analysis in an attempt to identify the pattern of cognitive factors associated to self-care behavior among the patients. Among all the participants, the majority were male (78.9%), married (85.6%), and older than 50 (64%). The mean age of the patients was 53.2 ± 12.5 with the range of 20 to 79 years. The results of each stage in regression-based path analysis were as follow:

Applying Spearman correlation coefficient test, we found statistically significant correlations between all the HBM variables and self-care behaviors. The range of *r* coefficient was from 0.310 (for the relationship between self-efficacy and self-care behaviors) to 0.610 (for the relationship between knowledge and self-care behaviors). The step 1 of the linear regression analysis was conducted to predict self-care behaviors. The results are presented in Table 2, level 1.

The step 2 linear regression showed the variables that significantly predicted self-care behaviors (knowledge *P* = 0.000, β = 0.399; perceived susceptibility *P* = 0.042, β = 0.158; and perceived barriers *P* = 0.000, β = 0.315). These 3 variables were again regressed on self-care behaviors to determine the strongest predictor for continuing the path. The strongest predictor was found to be knowledge (β= 0.422).

As the other remaining variables of the model, as independent variables, were regressed on knowledge, perceived barrier was found to be the only significant predictor (β = 0.250) (Table 2, level 3). At the step 4, perceived barrier, as the significant predictor in the previous step, was considered as the dependent variable. In this step, 2 variables (perceived susceptibility β= 0.202 and self-efficacy β= 207) were significant predictors of the dependent variable (Table 2, level 4).

At the step 5, the stronger predictor in the previous step, self-efficacy, was considered as the dependent variable, and after regressing all the other remaining variables of the model, as independent variables, on self-efficacy (Table 2, level 5), the only significant predictor was found to be perceived susceptibility (β= 0.322). At the step 6, perceived susceptibility was significantly predicted by 2 dependent variables (Perceived severity β= 0.285 and locus of control β= 0.484) (Table 2, level 6).

At the step 7, locus of control, as the strongest predictor in previous step, was considered as the dependent variable and the regression was run. We found perceived severity (β= 0.318) as the only significant predictor for locus of control (Table 2, level 7). At the step 8, perceived severity, as the only predictor in the previous step, was considered as the dependent variable. All the 3 remaining variables of the model, as independent variables, were regressed on perceived severity (Table 2, level 8) and we found 2 variables (perceived threat β= 0.190 and perceived benefits β= 0.525) as significant predictors of the dependent variable.

At the step 9, perceived benefits, as the strongest predictor in previous step, was considered as the dependent variable. The 2 remaining variables of the model, as independent variables, were regressed on perceived benefits (Table 2, level 9). The significant predictor was found to be perceived threat (β= 0.454). At the step 10, perceived threat, as the only predictor in the previous step, was considered as the dependent variable. Cues to action as the only remaining variable of the model was regressed on perceived threat (Table 2, level 10) and the regression-based path analysis terminated in this step.

Table 3 illustrates the direct, indirect and total effects of independent predictors on self-care behaviors. This table shows the positive direct effects of knowledge (r = 0.422), perceived barriers (r = 0.420) and perceived susceptibility (r = 0.208) on self-care behaviors. The other independent variables had indirect effects on self-care behaviors.

**Figure 1:**
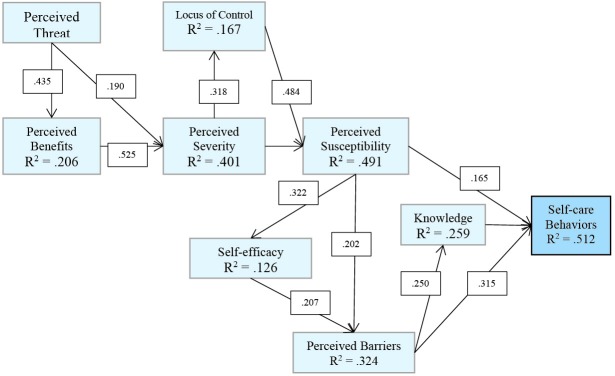
Regression-based path of Health Belief Model constructs appropriate to the HF patients participated in the present study.

Figure 1 illustrates the regression-based path of HBM constructs appropriate to the HF patients in the present study. As there is shown in the figure, knowledge, perceived susceptibility and perceived barriers had direct effects on self-care behaviors. Among these 3 variables, knowledge had the highest effect on the dependent variable. Perceived benefits, self-efficacy, perceived severity, perceived threat, locus of control and cues to action had indirect effects on self-care behaviors through the first 3 variables. The lowest value of effects on self-care behaviors was for cues to action.

## Discussion

Our aim in the present study was to determine the direct and indirect associations of the HBM-based cognitive factors with self-care behaviors among patients with HF. The regression-based path analysis showed positive direct relations of knowledge, perceived barriers and perceived susceptibility with self-care behaviors. We found these 3 variables as the strongest predictors for self-care behaviors, which mean that by increasing perceived susceptibility and knowledge, and decreasing perceived barriers, it may be expected to increase the level of performing self-care behaviors by about 51%. These findings were somewhat consistent with those found in the original study,^18^ within which the main predictors of the behaviors were knowledge, perceived threat and perceived benefits. In the linear regression model of the original study, perceived barriers, self-efficacy and severity as well as locus of control were not significant predictors of the behaviors. In the present study, however, these constructs were found to have either direct or indirect impacts on self-care behaviors. These findings are consistent with those reported by previous studies.^22,23^ We also found perceived barriers as one of the strongest predictors for performing self-care behaviors among the patients. Similarly, Moshki et al in a previous study found that reducing the barriers of cardiovascular diseases’ preventive behaviors may promote the efforts of the patients in performing the behaviors.^24^ Robinson^25^ and Tanner-Smith and Brown^26^ also reported perceived barriers as the strongest predictor for disease preventive behaviors.

The HBM was found to be useful in determining the direct and indirect associations of cognitive determinants with self-care behaviors among the patients with HF. This psychological model not only supports the researchers to realize the determinants of health behavior but also helps them in finding the potentially modifiable factors of the behavior.^27^ Among the HF patients in the present study, for instance, the recommendations provided to the patients by the physicians and health care providers (cues to action) may lead the patients to increase their knowledge. Higher levels of knowledge may lead them to higher levels of perceived susceptibility and severity toward the disease. Such high level of perception on the disease and its outcomes may enforce the patients to find strategies for overcoming the barriers of performing self-care behaviors. In this main path, the mediating role of some other cognitive factors like perceived self-efficacy, locus of control and perceived benefits may not be ignored.

Self-efficacy, for example, had a significant association with perceived barriers. It means that in the case of intervention to diminish the level of perceived barriers for performing HF self-care behaviors, the patients’ belief on their ability to overcome the barriers of self-care should be considered as a main domain of the intervention. Moreover, based on our results, locus of control mediated the relation of perceived severity with perceived susceptibility and self-care behaviors. As an interpretation, when a HF patient perceives the severity of the disease, he may consider either a high or low level of control on managing the disease. If a HF patient considers the locus of control for his disease as internal, he may consider himself as a core agent to control the disease. In contrast, if he considers the locus of disease control as external, he may consider some factors other than himself as core categories to control the disease. Therefore, he may not have an optimal level of effort to manage his disease. Now, if he considers the locus of disease control as internal, he may find himself more susceptible toward the disease outcomes and thus may be motivated to comply with the self-care behaviors.

Considering the total effect of the independent variables on self-care behaviors in the present study, knowledge had the greatest effect and was the most significant predictor for self-care behavior in HF patients. This finding shows knowledge as the most influential element on performing such behaviors among the patients, which is similar with those reported in previous studies.^3,28^ In a study among women, Ali et al found that susceptibility toward CHD outcomes, knowledge on the risk factors of CHD, and general health motivation explained 76% of the variance in performing CHD preventive behaviors.^29^ Therefore, promoting knowledge should still be considered as one of the core categories while designing health promotion programs aiming at the self-management of HF among the patients.

Self-care behaviors may be adopted by the patients if perceived barriers to perform the behaviors are alleviated, and if the patients’ knowledge on and perceived susceptibility toward the disease and its outcomes are improved. Based on our results, important factors in decreasing the level of perceived barriers towards self-care performance were self-efficacy and perceived susceptibility. Perceived susceptibility should be considered as a core category in adopting the behaviors among patients with HF, considering its role in promoting self-efficacy and alleviating perceived barriers. Albert Bandura offered self-efficacy as a perception that may be contributed to cognitive development and practice,^30^ and supposed its influence through cognitive, motivational, impressive, and choice processes. Kulviwat et al also proposed self-efficacy as a main antecedent of cognition and affect while evaluating the utility and facility of adopting new behaviors.^8^ On the other hand, perceived susceptibility toward the disease and its outcomes may be promoted if the patients’ perception on the severity of the disease and its outcomes and the locus of control are also improved.

### Limitation

This study was a secondary analysis on a dataset obtained through a quasi-experimental research. We used the pre-intervention data which was collected from a convenient sample of HF patients. Therefore, causal inferences are warranted.

## Conclusion

Our findings may be useful in designing interventional programs aiming at self-care behaviors promotion among HF patients. HBM was found to be helpful in understanding the direct and indirect associations of cognitive determinants with self-care behaviors among these patients. It may also be suggested for health promotion planning among these patients. Based on this challenging path analysis, HF patients’ knowledge and perceived barriers and susceptibility are suggested as the most core categories while designing HF educational programs. Although perceived severity, self-efficacy, benefits and threat as well as locus of control were not in direct associations with self-care behaviors, their indirect role on performing these behaviors should not be neglected in patient education programs. Practitioners, nurses and healthcare providers with a better understanding on the associations between these psychological determinants of self-care behaviors may design more properly informed stage-specific educational interventions aiming to foster disease management behaviors among HF patients.

## Ethical approval

All procedures performed in the study involving human participants were in accordance with the ethical standards of the institutional and/or national research committee and with the 1964 Helsinki declaration and its later amendments or comparable ethical standards. The original study, based on which this analysis was conducted, was approved by the Research Ethical Committee of Yazd University of Medical Sciences, Yazd, Iran. Written informed consent was obtained from all subjects. Informed consent was obtained from all individual participants included in the study.

## Competing interests

The authors declare no conflicts of interest, both financial and non-financial, for this study.

## Funding

No fund was received to conduct this study.

## Authors’ contributions

HN and JS collected the initial data. HN and HM were involved in the conception of the study, performed the analyses, and drafted the manuscript. HN, HR, and ZR were involved in the interpretation of the results from the analyses. HR and ZR assisted in drafting and revising the manuscript.

## Acknowledgments

The authors thank the initial research team who provided us with the data.

**Table 1:** Scales, number of items, reliability coefficients in the pilot and final sample, and possible ranges of the HBM-based constructs

**Variables**	**Score**	**Number of items**	**Possible range**	**Cronbach α**
HF self-care behavior scale	0-60	15	0-4	0.81
Knowledge	0-12	6	0-2	0.78
Susceptibility	0-20	5	0-4	0.89
Severity	0-24	6	0-4	0.82
Perceived threat	0-8	2	0-4	0.71
Perceived barriers	0-28	7	0-4	0.86
Perceived benefits	0-24	6	0-4	0.80
Locus of control	0-24	6	0-4	0.91
Self-efficacy	0-18	6	0-3	0.93

**Table 2:** Regression-based path analysis of HBM model constructs to predict self-care behaviors among HF patients

**Level**	**Independent variables**	**Standardized Beta**	***P*** ** value**	**R** ^ 2 ^	**Dependent variables**
1	Knowledge	0.399	0.000	0.527	Self-care Behaviors
	Perceived benefit	0.118	0.099		
	Severity	-0.039	0.610		
	Susceptibility	0.158	0.042		
	Locus of control	-0.42	0.542		
	Perceived threat	0.044	0.480		
	Perceived barriers	0.282	0.000		
	Self-efficacy	0.050	0.385		
2	Knowledge	0.422	0.000	0.512	Self-care Behaviors
	Susceptibility	0.165	0.007		
	Perceived barriers	0.315	0.000		
3	Susceptibility	0.036	0.708	0.259	Knowledge
	Perceived barriers	0.250	0.002		
	Perceived benefit	0.116	0.192		
	Perceived severity	-0.053	0.563		
	Locus of control	0.154	0.074		
	Perceived threat	0.152	0.051		
	Self-efficacy	0. 093	0.201		
	Cues to action	-0.085	0.209		
4	Susceptibility	0.202	0.027	0.324	Perceived barriers
	Perceived benefit	0.143	0.087		
	Perceived severity	0.020	0.821		
	Locus of control	0.153	0.060		
	Perceived threat	0.105	0.154		
	Self-efficacy	0.207	0.002		
	Cues to action	0.053	0.408		
5	Susceptibility	0.322	0.001	0.126	Self-efficacy
	Perceived benefit	0.139	0.139		
	Perceived severity	-0.100	0.317		
	Locus of control	-0.034	0.712		
	Perceived threat	-0.007	0.935		
	Cues to action	0.130	0.073		
6	Perceived benefit	0.012	0.867	0.491	Perceived susceptibility
	Perceived severity	0.285	0.000		
	Locus of control	0.484	0.000		
	Perceived threat	0.114	0.071		
	Cues to action	0.014	0.804		
7	Perceived benefit	0.147	0.106	0.167	Locus of control
	Perceived severity	0.318	0.000		
	Perceived threat	-0.047	0.558		
	Cues to action	0.069	0.324		
8	Perceived benefit	0.525	0.000	0.401	Perceived severity
	Perceived threat	0.190	0.004		
	Cues to action	-0.083	0.161		
9	Perceived threat	0.454	0.000	0.206	Perceived benefit
	Cues to action	-0.003	0.960		
10	Cues to action	0.125	0.095	0.149	Perceived threat

**Table 3:** Direct, indirect and total effects of independent predictors on self-care behaviors based on regression-based path analysis

**Independent variables**	Direct effect*	Indirect effect**	**Total effect** ^***^	**Dependent variable**
Knowledge	.422		0.422	Self-care behaviors
Perceived susceptibility	.165	(.322×.207×.250×.422) + (.202×.250×.422) + (.036×.422) = .043	0.208	
Perceived barriers	.315	.250×.422=.105 (effect of perceived barriers on knowledge× effect of knowledge on self-care behaviors)	0.420		
Self-efficacy	_	(.207×.250×.315) + (.093×.442)	0.057	
Perceived severity	_	(.318×.484×.322×.207×.250×.422) + (.285×.322×.207×.250×.422) + (-.100×.207×.250×.422) + (.020×.250×.422) + (-.053×.422)	0.178	
Locus of control	_	(.484×.322×.207×.250×.422) + (-.034×.207×.250×.422)+(.153 ×.250×.422) + (.154 ×.422) =	0.085	
Perceived threat	_	(.152×.422)+ (.105×.250×.422) +( -.007×.207×.250×.422) +(.114×.322×.207×.250×.422)+ ( -.047×.484×.322×.207×.250×.422)+(.190×.318×.484×.322×.207×.250×.422)+(.454×.525×.318×.484×.322×.207×.250×.422)	0.073	
Perceived benefits	_	(.525×.318×.484×.322×.207×.250×.422)+(.147×.484×.322×.207×.250×.422)+( .012×.322×.207×.250×.422)+( .139×.207×.250×.422)+( .143×.250×.422)+( .116×.422)	0.172	
Cues to action	_	(.525×.318×.484×.322×.207×.250×.422)+(.147×.484×.322×.207×.250×.422)+( .012×.322×.207×.250×.422)+( .139×.207×.250×.422)+( .143×.250×.422)+( .116×.422) (.228×.315)+(.182×.165)+(.182×.393×.419×.422)+(.182×.393×.315)+(.053×.250×.422)+ (-.085×.442)+(.130×.207×.250×.422)+(.014×.322×.207×.250×.422)+(.069×.484×.322×.207 .003×.525×.318×.484×.322×.207×.250×.422)+(.125×.454×.525×.318×.484×.322×.207×.250×.422)	0.192	

* The direct effect means that the independent variable has direct effect on self -care behaviors. ** The indirect effect means that the independent variable has indirect effect on self-care behaviors through other variables. *** The total effect is the sum of direct and indirect effects of independent variable on dependent variable (self-care behaviors).
